# SLiMPrints: conservation-based discovery of functional motif fingerprints in intrinsically disordered protein regions

**DOI:** 10.1093/nar/gks854

**Published:** 2012-09-12

**Authors:** Norman E. Davey, Joanne L. Cowan, Denis C. Shields, Toby J. Gibson, Mark J. Coldwell, Richard J. Edwards

**Affiliations:** ^1^Structural and Computational Biology Unit, European Molecular Biology Laboratory, Heidelberg, Baden-Württemberg 69117, Germany, ^2^Centre for Biological Sciences, University of Southampton, Southampton SO17 1BJ, UK, ^3^UCD Complex and Adaptive Systems Laboratory, University College Dublin, Dublin 4, Ireland, ^4^UCD Conway Institute and School of Medicine and Medical Sciences, University College Dublin, Dublin 4, Ireland and ^5^Institute for Life Sciences, University of Southampton, Southampton SO17 1BJ, UK

## Abstract

Large portions of higher eukaryotic proteomes are intrinsically disordered, and abundant evidence suggests that these unstructured regions of proteins are rich in regulatory interaction interfaces. A major class of disordered interaction interfaces are the compact and degenerate modules known as short linear motifs (SLiMs). As a result of the difficulties associated with the experimental identification and validation of SLiMs, our understanding of these modules is limited, advocating the use of computational methods to focus experimental discovery. This article evaluates the use of evolutionary conservation as a discriminatory technique for motif discovery. A statistical framework is introduced to assess the significance of relatively conserved residues, quantifying the likelihood a residue will have a particular level of conservation given the conservation of the surrounding residues. The framework is expanded to assess the significance of groupings of conserved residues, a metric that forms the basis of SLiMPrints (short linear motif fingerprints), a *de novo* motif discovery tool. SLiMPrints identifies relatively overconstrained proximal groupings of residues within intrinsically disordered regions, indicative of putatively functional motifs. Finally, the human proteome is analysed to create a set of highly conserved putative motif instances, including a novel site on translation initiation factor eIF2A that may regulate translation through binding of eIF4E.

## INTRODUCTION

During the past decade, there has been increasing focus on the role of intrinsically disordered polypeptide regions in protein functionality (1–4), resulting in a more complete understanding of the complex wiring of the interactome, and revealing an unexpected level of complexity and cooperativity (5). Short linear motifs (SLiMs) in particular are highly overrepresented in these regions, playing a vital regulatory role by acting as targeting signals, modification sites and ligand binding modules (6–8). SLiMs have extremely compact protein interaction interfaces [generally encoded by less than four major affinity and specificity determining residues within a stretch of 2–10 residues (9)], and this small footprint promotes high functional density. This property facilitates competitive and cooperative binding, allowing complex switches to evolve from a multiplicity of SLiMs, which can be regulated further by the modification state of the protein and local abundance of interaction partners (10–13). The limited size of the interfaces results in micromolar binding affinity for SLiM interactions, enabling the transient and reversible interactions necessary for many dynamic cellular binding events, such as those required for the rapid transmission of intracellular signals (14). Furthermore, SLiMs have an inherent evolutionary plasticity, allowing novel instances to evolve *de novo*, adding functionality and regulatory constraints to proteins, thus rewiring pathways, a property central to the evolvability of complex systems (15). This evolutionary mechanism promotes redundancy and introduces robustness (16); therefore, motifs often possess weak phenotypes so that malfunctioning motifs are rarely seen to be the primary cause of disease, although exceptions exist (17–19). However, this evolutionary plasticity also has drawbacks, as it renders motifs highly susceptible to mimicry by rapidly evolving pathogens that use them to hijack cellular processes (17,20).

The relatively weak phenotypic effects of most SLiM mutations can lead to difficulties in experimental discovery. Therefore, multiple computational approaches have been proposed to discover motifs in biological data, pinpointing sites likely to be functional SLiMs (21). The eukaryotic linear motif (ELM) (22) and Minimotif (8) servers identify regions of a protein matching regular expressions of known functional SLiMs, filtering matches on discriminatory attributes based on analysis of curated experimentally validated motifs; SLiMSearch (23) performs a similar task for user-defined SLiMs. Tools such as SLiMFinder (24,25) and Dilimot (26) use the same attributes to attempt novel SLiM discovery by identifying overrepresented convergently evolved motifs in interaction, localization or gene ontology data. More recently, *de novo* discovery methods acting on protein primary sequence, utilizing features of a motif that contrast with a disordered context as a pointer to functionality, have been suggested. For example, α-MoRF (27) uses a machine learning approach to identify stretches with the potential to adopt α-helices within regions of disorder; ANCHOR (28) applies biophysical principles to identify stretches of protein sequences that may fold when given stabilizing energy contributed by a globular partner; SLiMPred (29) uses machine learning to identify characteristic sequence features derived from known SLiM occurrences.

Because of the lack of constraints associated with the conservation of a stable globular fold, SLiMs are under weaker evolutionary constraints than structured domains. However, these short intrinsically disordered modules are often under strong functional constraint; therefore, functionally important residues within these motifs are more conserved than adjacent non-functional residues (9,30). As a post-processing step, conservation is often used for classification in motif discovery methods. Classifying putative SLiMs based on conservation has proved to be a good discriminator of motif functionality (31,32). Recent motif surveys have used these discriminators to classify motifs and discover novel instances of SH3-domain binding and KEN box motifs (33,34). Furthermore, pre-processing by protein masking based on evolutionary constraint has also been shown to increase the ability of discovery methods to return previously experimentally validated functional motifs (30), which has recently been exploited in proteome-wide prediction of human SLiMs (35).

Homology-based methods revolutionized the discovery of globular domains resulting in an explosion in the number of known globular domains (36,37). However, because of the length and degeneracy of SLiMs, these methods are unsuitable for motif discovery. Intriguingly, the human proteome is punctuated by regions of relatively high conservation against a background of evolutionary drift in intrinsically disordered stretches of proteins that are indicative of a functional SLiM (30,35). This functional constraint is often clearly visible in multiple sequence alignments as an island of conservation in otherwise rapidly evolving regions, and it is often successfully used as a pointer by motif biologists attempting to discover novel motifs (38). However, simply scanning the alignments by eye is problematic, as we are accustomed to finding patterns, and homing in on what seems most interesting, but manual scanning is less useful to guess how unlikely the observed regions are. Recently, efforts have been made to automate this approach, using profile–profile comparison to discover shared motifs in distantly related viral proteins (39) and using hidden Markov models to computationally identify short stretches of conserved disordered regions in the yeast proteome (40). In this article, we tackle the problem of rapidly and robustly establishing the statistical significance of the relative conservation of small clusters of conserved residues within a disordered region. We also introduce a *de novo* motif discovery method, SLiMPrints (short linear motif fingerprints), to identify putative functional motifs in the primary sequence using these relative conservation statistics. The SLiMPrints method is applied to the human proteome to produce a database of highly conserved motif-like groupings of proximal residues in disordered regions.

## MATERIALS AND METHODS

### Islands of conservation in rapidly evolving disordered regions

**Figure 1.**
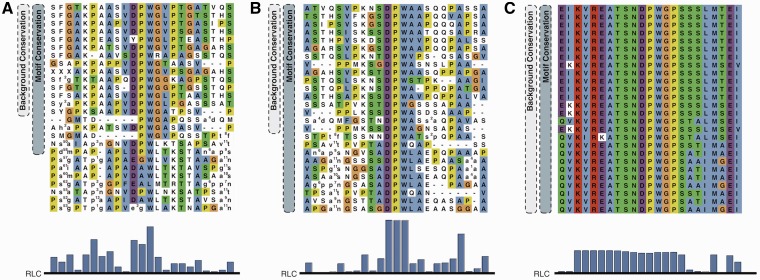
(**A–C**) are sections of human Epsin 2 containing functionally important residues matching the regular expression for the AP2-binding motif DPW aligned against a selection of vertebrate Epsin 2 orthologues. Pairs of lower case letters denote amino acids flanking a region inserted compared with human Epsin 2 and the number specifies the length of the insertion. The alignment is coloured using the Clustal colouring scheme. (A and B) are known functional AP2-binding motifs and (C) is part of the N-Terminal ENTH domain. Lower panel: RLC scores for the section of the alignment (see ‘Materials and Methods’ section for a description of the RLC scoring scheme).

Figure 1 shows three functionally important stretches in Epsin 2 matching the DPW (Asp-Pro-Trp) regular expression of an AP2 binding motif (ELM entry LIG_AP2alpha_2) (41). Panel A is more conserved than its surrounding residues, yet not to such a degree that would suggest that the motif stands out as being under strong functional constraint. In contrast, the DPW motif in panel B permits more confidence in the assumption that the motif is functional because of conservation in a wide range of species, despite residing in a region with otherwise high mutation rates. Finally, the DPW pattern in panel C occurs within an ENTH domain and is under structural and functional constraints, as are its neighbouring regions, and the motif cannot be discriminated from surrounding residues based on conservation. In this article, we aim to statistically quantify the conservation of residues and motifs compared with their flanking regions and investigate discriminatory ability of these statistics to identify islands of conservation (similar to Figure 1, panel B) for ranking and discovery of putative SLiM instances.

### Benchmarking and human proteome data sets

The benchmarking data set for the analysis consists of a set of SLiM instances from the ELM database (March 2012; Supplementary Table S1) (22), a gold standard curated collection of experimentally validated motifs. The data set contains 1885 motif instances in 1234 proteins. The residues of these 1234 proteins were defined as ‘ELM residues’ for a defined position (fixed or degenerate, but not wild card) in an annotated ELM occurrence and as ‘non-ELM residues’ for the remaining residues. The human proteome data set consists of 20 253 reviewed proteins from the UniProt database (November 2011) (42).

### Orthologue alignment construction

Multiple sequence alignments of least divergent orthologues were constructed for each protein in the benchmarking data sets using the GOPHER algorithm (43) against a database of EnsEMBL metazoan (plus *Saccharomyces cerevisiae*) genomes (release 59) (44). To maximize proteome coverage while minimizing redundancy, a data set consisting of one protein sequence per protein-coding gene was constructed as previously described (35). Homologues for each protein sequence were identified using a BLAST search (45), and orthologues were predicted using default GOPHER parameters. Multiple sequence alignments for sets of orthologues were generated using MUSCLE (46). As a result of their complex (47) and rapid evolution (48), disordered regions are notoriously difficult to align (49). As the quality of the alignment is reflected in the quality of the conservation score, the orthologue alignments were processed to remove potential biases. Long branches are pruned as described by Chica *et al.* (32), to remove the contribution to the conservation score of stochastically aligned residues and low-complexity regions in highly divergent proteins. Only proteins that have residues aligned to the query sequence, regardless of physicochemical similarity, for >80% of the length of the query sequence are retained. Alignments with orthologues in <10 metazoan species after the pruning and filtering steps were not considered.

### Relative local conservation

Simple column based conservation metrics calculate conservation scores for a residue solely based on information from one column, and therefore cannot measure the attributes associated with islands of functional constraint indicative of a putatively functional SLiM. However, relative local conservation (RLC) (30), a conservation metric for scoring the constraint on a residue relative to a window of adjacent residues, allows such regions to be pinpointed. The RLC scoring scheme provides the basis for the motif discovery algorithm presented in this article; hence, it will be described again briefly in the following section, highlighting the improvements made to the scheme to allow efficient and accurate prediction of relatively overconstrained residues from protein alignments.

The residue conservation score, C*_i_*, for each column i of the alignment is calculated. As the method functions independently of the scoring scheme, in theory, any conservation-scoring scheme can be used, provided a model can be proposed for the background distribution of residues under no constraint. In this analysis, we use *CS* (32), a tree-weighted information content-based column score. The score is calculated based on identity, as the consideration of conservative substitutions markedly reduced the statistical power of the method (data not shown). The calculation of a relative conservation score requires a background conservation level for comparison. In this analysis, the background conservation level is calculated as the mean conservation of the flanking residues of the residue of interest. Considering only the constraints imposed on local residues negates the bias introduced by the disparity in conservation levels between globular and intrinsically disordered regions and the obvious effect this disparity, in association with varying proportions of globularity in a protein, would have on the mean conservation of a protein. A mean background conservation score is calculated across a window spanning *N* residues on either side of the residue *i*, raw residue conservation scores of each residue in a protein are converted to an RLC score by subtracting the mean background conservation across the appropriate window, and the RLC score is normalized by dividing by the standard deviation of conservation scores across the window (Equation 1). For this study, *N* was 30, yielding a window spanning from 30 residues upstream to 30 residues downstream of each amino acid.
(1)
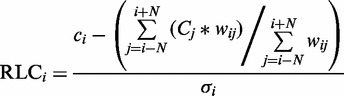



Calculation of the RLC score for residue *i* in a protein sequence. *N* is the number of residues either side of residue *i*, the window for the background conservation comparison. *w_ij_* is the weight applied to the residue *j* when calculating the RLC for residue *i. σ_i_* is the standard deviation of the conservation values (*C*) across the same window of residues used to generate the mean background local conservation score. When no weighting is used, or all residues have the same disorder score, the residue weighting (see Equation 2) *w_ij_* becomes 1.

The RLC scoring scheme is weighted to offset the effect of differences in conservation between intrinsically disordered and globular regions within a window. This bias can be particularly strong at order/disorder boundaries, as these regions are often fuzzily defined by disorder predictors, which can result in strong structural constraints (i.e. those important for correct folding of a globular domain), being mistaken for constraints on specificity and affinity determining residues within interaction interfaces in non-globular regions. A continuous weighted scheme allows residues within the window with a similar tendency to disorder to the residue being considered to contribute more strongly to the mean background conservation score of that residue. The scheme is also asymmetric, lessening the contribution of residues of non-similar disorder state in a manner that residues with a higher tendency to order (those more likely to be under structural constraints than the residue of interest) influence the RLC score less than residues with a higher tendency to disorder (Equation 2).
(2)
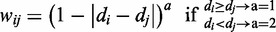

where *w_ij_* is the weight applied to the residue *j* when calculating the RLC for residue *i. a* is a value that controls the strictness of the weighting. *d_x_* is the IUPred disorder score at residue *x*.

### Masking of motif deficient regions

Large regions of protein space are deficient in motifs, and masking these regions is a common technique to improve the statistical power of motif discovery methods (26,30,32). For this analysis, Pfam annotated domains (37), transmembrane regions and extracellular regions from UniProt (42) and residues with an IUPred score <0.3 (50) are masked before the motif discovery step of the method. Several difficult-to-handle regions observed to introduce a bias to the conservation score were not considered and removed from the analysis: regions with homogeneous conservation (those with a *CS* standard deviation for a window <0.01), ‘gappy’ weakly aligned regions (>25% of the positions of a column of the alignment, after long branch pruning, are gaps), ‘short’ unmasked stretches of the protein <10 amino acids in length.

### Probability of relative local conservation scores

Benchmarking a probabilistic framework to calculate the likelihood that a residue will occur with a given RLC by chance requires a background data set that models the distribution of RLC scores for residues under no functional constraint. For this analysis, we followed the simplified hypothesis that residues in disordered regions [as defined by IUPred score >0.3 (50)], but not in annotated ELM instances, are under no functional constraint. The incomplete coverage of ELM means that many functional residues in disordered regions will be annotated as background non-ELM residues for this analysis. Furthermore, residues flanking annotated motifs contributing to the motif binding, but not directly in the interface, may also be under functional constraint and still defined as non-ELM residues. However, the strong enrichment for functional residues in the ELM residues set still makes this a valid benchmarking exercise. RLC scores were calculated for unmasked ELM residues and non-ELM residues in the ELM benchmarking data set. The mean RLC of non-ELM residues (280 994) is −0.06, which is close to the expected mean of 0. As expected for a stochastically occurring RLC distribution, approximately half of the background residues (46.9% in the ELM benchmarking data set, 49.1% in the human proteome data set) have RLC scores >0. Conversely, the unmasked ELM residues (3728 in 1002 ELM) are strongly enriched for positive RLC scores (78.5% of these residues have RLC >0, and the mean RLC score of these residues is 0.78).

**Figure 2.**
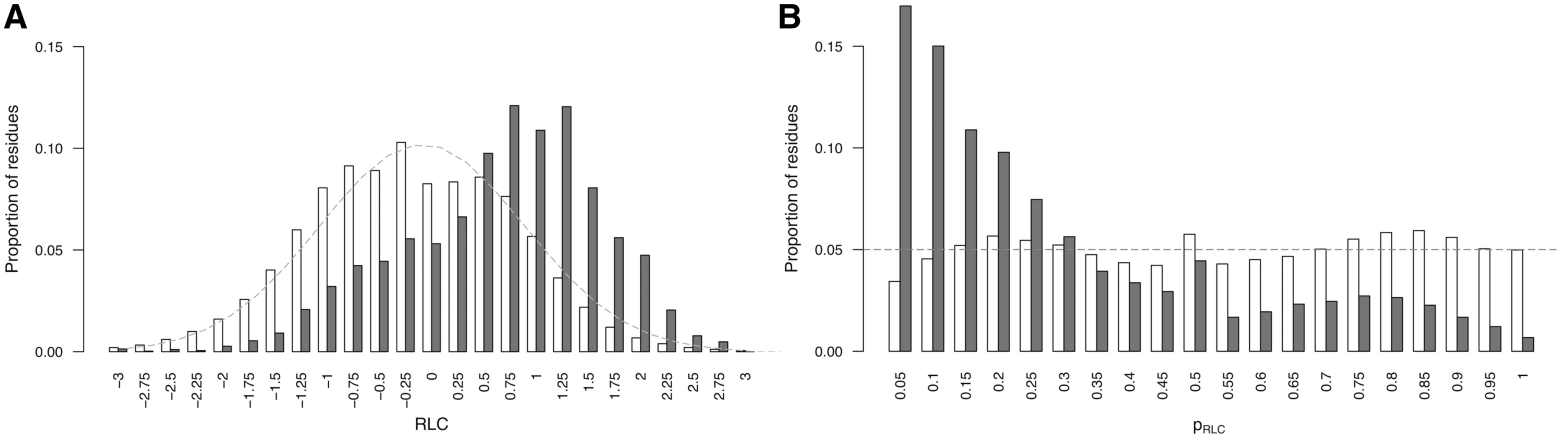
Comparison of ‘non-ELM residues’ residues (white) against ‘ELM residues’ (grey) from the benchmarking data set from the ELM resource. (**A**) RLC value comparison, grey dashed line shows a Gaussian distribution (µ = 0, σ = 1). RLC values on *x*-axis are lower limits of bins of size 0.25. (**B**) *p*_RLC_ value comparison, grey dashed line shows a uniform distribution. *p*_RLC_ values on *x*-axis are lower limits of bins of size 0.05.

The distribution of RLC scores for unmasked non-ELM residues in the benchmarking data set can be roughly approximated by a Gaussian distribution with a mean 0 and standard deviation, σ, of 1 (Figure 2A). There is a slight shift towards negative values, this is likely to be because of the presence of highly conserved ELM instances that raise the background conservation score (and therefore lower the RLC scores) of nearby non-ELM residues. The scoring scheme assumes that the residues in the background data set are under no constraint, whereas the ELM benchmarking data set is obviously enriched for constrained residues. This is supported by the observation that background distribution for human proteome data set shows a much weaker bias. Assuming a Gaussian distribution for RLC values allows the probability of a given RLC value to be calculated simply using the Gaussian cumulative distribution function (Equation 3). Overall, the heuristic approach provides a satisfactory approximation for the probability of a disordered residue occurring with a given RLC or greater by chance, as is illustrated by the similarity of *p*_RLC_ to a uniform distribution (a true statistical *P*-value should be uniformly distributed) for the non-ELM residues (Figure 2B). However, we would stress that these *P*-values are dependent on a number of model assumptions, and should be interpreted accordingly with some caution.
(3)
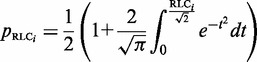

Using the heuristic assumption of normality allows the probability of a residue occurring with a given RLC or more, *p*_RLC_, from a derivation of the Gaussian cumulative distribution function.

### Motif discovery hypothesis

Given the simplified hypothesis that intrinsic disorder is under no evolutionary constraint, adjacent residues can be considered to be evolving independently; therefore, residues with stochastically high-RLC scores should be spread out randomly throughout the disordered regions of a protein. However, functional motif modules within disordered regions, because of their linear nature and functional importance, should be small proximal groupings of relatively overconstrained residues (9). Identification of such groupings would allow putatively functional motifs to be discovered. We call such groupings ‘SLiMPrints’, and the SLiMPrints prediction method is described later in the text.

### Motif building

**Figure 3.**
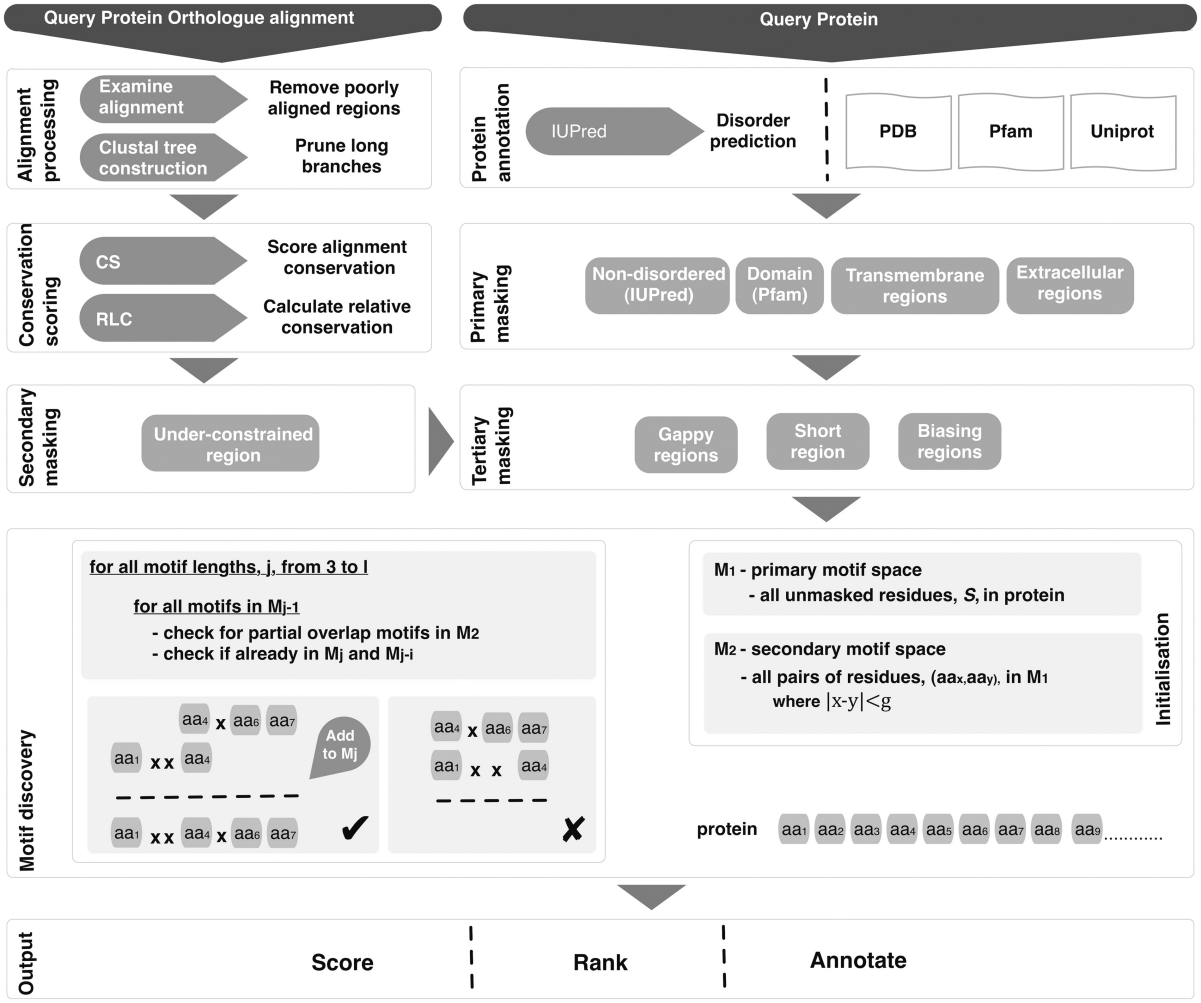
Schema of the SLiMPrints method.

Given the maximum allowable number of fixed positions, *l*, in a returned motif and the maximum length of wild card (any residue) ‘gaps’ allowed between fixed positions *g,* a motif search space, *M*, can be defined based on the attributes of known functional motifs (for this analysis *l* = 5 and *g* = 2). Restricting the search space to proximal residues minimizes the likelihood of randomly occurring high-scoring groups of residues; therefore, maximizes the probability of discovering a functional motif against a background of randomly constrained residues. A simple motif-building algorithm (Figure 3) can be used to search for motifs that are compatible with the motif search space. The initial step takes all residues with an RLC above *c*, a conservation cut-off (default *c* ≥ 0), to define the residue search space and initial motif search space, *S* and *M_1_* (motif search space of length 1), respectively. For each motif length *i* (up to the maximum length *l*), the motif space *M_i_* is established by extending the motif search space *M_i-__1_*. Each motif, *m*, in *M_i-__1_* is taken in turn and expanded by the addition of each residue, *r*, (where *r* ∈ *S*) for which the offset of *r* is within the gap length *g* of either side of motif *m*. Only deletions and insertions less than *d* (default *d* = 2) residues long within the region of the alignment spanned by the motif and matching the motif are tolerated; no constraint is imposed on such regions not matching the motif. Motifs with significantly differing conservation scores for the defined residues (*p*_RLC_ variance >0.05), indicative of anchored residues in an alignment, are discarded.

### Motif scoring

**Figure 4.**
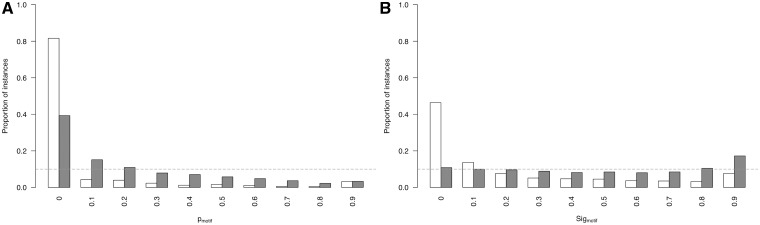
Comparison of the *p*_motif_ and *Sig*_motif_ score distributions for ‘non ELM residues’ residues (grey) against ‘ELM residues’ (white). (**A**) *p*_motif_ score distributions. *p*_motif_ values on *x*-axis are lower limits of bins of size 0.1. (**B**) *Sig*_motif_ score distributions. *Sig*_motif_ values on *x*-axis are lower limits of bins of size 0.1.

Equation 4 introduces a metric to calculate the likelihood of a group of highly conserved proximal residues. The relative conservation probability of a motif, *p*_motif_, determines the probability of each defined residue within a motif having its given RLC or higher by chance (Figure 4A). However, *p*_motif_ cannot be used to compare motifs with differing numbers of fixed positions, as longer motifs generally have lower *p*_motif_ scores, and it is not uniformly distributed as a true *P*-value. A significance value, *Sig*_motif_, representing the probability of a given motif having that *p*_motif_ value or higher by chance, can then be calculated for the motifs *p*_motif_ value using the cumulative distribution function of the uniform product distribution (Equation 5). This *Sig*_motif_ closely follows a uniform distribution (Figure 4B) and can be used as a heuristic statistical measure to quantify the likelihood of a grouping of highly conserved residues in a disordered region.
(4)
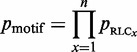

where *n* is the number of defined positions in the motif and *p*_RLC_*_x_*is the probability of the *x*-th residue of the motif occurring with a given RLC or more.
(5)
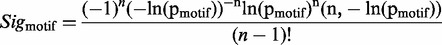

where *Sig*_motif_, the probability of a given motif having that *p*_motif_ value or higher by chance, calculated as the cumulative distribution function of the uniform product distribution, i.e. the distribution of the product of *n* uniform distributions. Where *n* is the number of non-wild card positions in the motif, *p*_motif_ is the relative conservation probability of a motif (Equation 4) and Γ is the incomplete gamma function.

### eIF2A–eIF4E binding experiments

For all experiments, HeLa cells were maintained in high-glucose Dulbecco’s modified Eagle’s medium supplemented with Glutamax (Life Technologies) and 10% fetal bovine serum in standard tissue cultureware at 37°C and in a 5% CO_2_ atmosphere. Cells were seeded 24 h before transfection with GeneJuice (Merck) according to the manufacturer’s protocol and were harvested 48 h after transfection. Cells were harvested for either co-immunoprecipitation using the Pierce Co-Immunoprecipitation kit (Thermo Scientific), or to make an S10 lysate ready for precipitation with 7-methyl-Guanosine triphosphate (GTP) Sepharose 4B (GE Healthcare), as per our previous protocol (51). Antibodies to the FLAG epitope (Clone M2, affinity purified) and to eIF2A were obtained from Sigma-Aldrich and Bethyl Laboratories, respectively. The eIF4E antibody was a kind gift from Prof. Simon Morley (University of Sussex, UK).

A plasmid containing a complementary DNA IMAGE clone of eIF2A corresponding to GenBank accession BC011885 was obtained from Open Biosystems. The primers eIF2A_CDS_F (GGTA aagctt ATGGCGCCGTCCACGCCGCT) and eIF2A_CDS_R (ATGG ctcgag TTAAATACCCAATTCCA) were used to amplify the open reading frame and incorporate HindIII and XhoI restriction enzyme sites into the respective 5′ and 3′ ends of the amplification product. After restriction digest, the sequence was subcloned into a pcDNA3.1(+) plasmid (Invitrogen), which had been previously engineered to include an N-terminal FLAG tag [as per the N-terminally myc-tagged plasmid discussed in (52)], to which the eIF2A open reading frame was fused. This plasmid was subsequently used as a template for Quikchange site-directed mutagenesis (Agilent Technologies) using the following primers:

YRPPALR_ ARAAALA_F gaggaacctaaagttgcaacagctgctagagccgcagctttagcaaataaaccaatcaccaattccaa; YRPPALR_ ARAAALA_R ttggaattggtgattggtttatttgctaaagctgcggctctagcagctgttgcaactttaggttcctc; YRPPALR_AAAAAAA_F gaagtacccaatgaggaacctaaagttgcaacagctgctgcagccgcagctgcagcaaataaaccaatcaccaattccaaattgcatgaaga; YRPPALR_AAAAAAA_R tcttcatgcaatttggaattggtgattggtttatttgctgcagctgcggctgcagcagctgttgcaactttaggttcctcattgggtacttc.

The sequence of all plasmids was confirmed by automated sequencing.

### Availability

Data sets used in the ELM benchmarking and human proteome analysis are available at http://bioware.ucd.ie/∼slimdb/. A webserver for the SLiMPrints method is available at http://bioware.ucd.ie/slimprints.html.

## RESULTS

### RLC-based probability measures as discriminators for functionality

**Figure 5.**
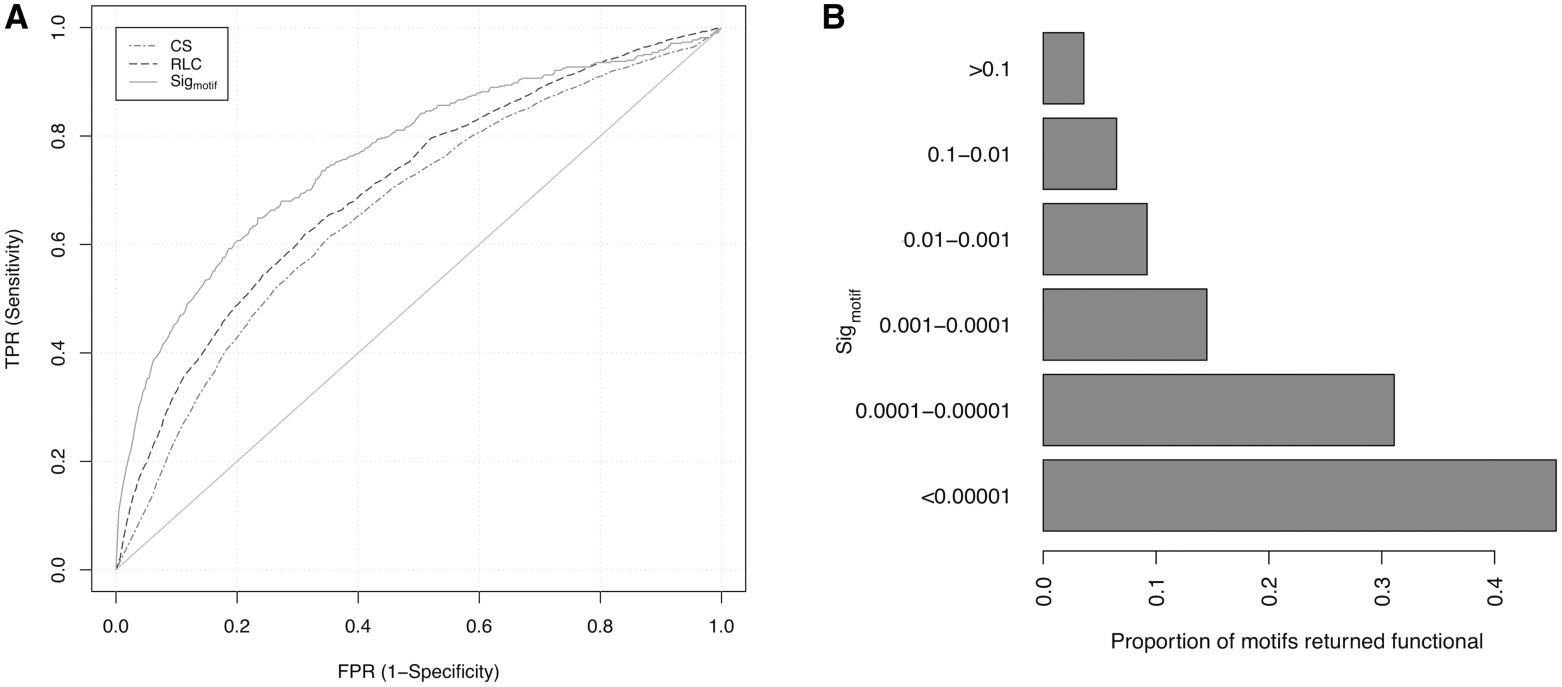
(**A**) ROC curve of *CS* (dashed and dotted), RLC (dashed) and *Sig*_motif _(solid) metrics. (**B**) Proportion of motifs returned at different *Sig*_motif_ scores that are experimentally validated functional motifs from the ELM database.

The ELM benchmarking data set was used to test the power of the *CS*, *p*_RLC _and the *Sig*_motif_ statistics to distinguish ELM and non-ELM residues, and the results are visualized as ROC (receiver operating characteristic) curve plots (Figure 5A). The upper left hand corner of a ROC curve plot is optimal, showing perfect discrimination between true positives (TPs) and false positives, whereas lines close to the diagonal suggest that a metric has no predictive power. Although absolute column-based conservation can clearly discriminate residues contained within ELMs (red line) compared with background residues (diagonal), considering the local conservation improves performance (blue line, *p*_RLC_). The clustering of motif residues (green line, *Sig*_motif_) clearly improves performance still further (calculated on the defined residues of the TP ELM instance against ELM regular expression hits in the non-ELM residues). Thus, it is not simply the relative conservation, but a metric that considers the grouping of more than one conserved residue that discriminates functional motifs most clearly.

### ELM benchmarking of SLiMPrints as a SLiM discovery method

The SLiMPrints method was applied to proteins of the ELM benchmarking data set (22) (see ‘Materials and Methods’ section), and returned motifs were analysed. A total of 1234 alignments containing 1885 ELM instances were tested (Supplementary Table S1). Of these, because of the strict filtering criteria, 883 instances were in regions not considered by the method: 330 instances were in regions defined as ‘globular’, 282 instances in 196 proteins had insufficient orthologues in the metazoan alignments, 67 were in regions defined as ‘gappy’, 23 were in ‘short’ regions and 8 were in extracellular regions. A total of 1002 ELM instances were contained in regions retained after the filtering step. Approximately 34 million overlapping motifs were considered by the method [estimated based on the number of residues in the search space (284 722) and the default motif attributes for allowable number of defined positions (5) and maximum gap length between defined positions (2)].

The ELM benchmarking data set returned 6391 distinct non-overlapping SLiMPrint motifs at a *Sig*_motif_ cut-off of 0.05, including 591 ELM motifs (Supplementary Table S2), i.e. 59.0% (591/1002) of the discoverable ELM motifs were ‘rediscovered’ in the benchmarking data set using solely their conservation fingerprint defined by *Sig*_motif_. In total, 9.2% (591/6391) of all motifs returned were overlapping experimentally validated functional ELMs. There is a strong and increasing enrichment of annotated ELMs as *Sig*_motif_ cut-offs decrease (Figure 5B), a desirable attribute for the discovery of low-hanging fruit in a whole proteome analysis. At *Sig*_motif_ scores <0.001, 18.9% (147/629) of the returned motifs were ELMs encompassing 14.7% of the annotated ELMs in the benchmarking data set, whereas at *Sig*_motif_ scores <0.00001, 45.5% (15/33) of the returned motifs were ELMs. Although 5800 of motifs returned were classified as non-ELM motifs because they did not overlap a region annotated as a functional motif in the ELM database, many of the high-scoring non-ELM motifs are experimentally validated functional motifs not annotated in the ELM database meaning that, in reality, the specificity of the method is far higher than this benchmarking suggests. For example, of the 18 motifs with *Sig*_motif_ scores <0.00001, but without ELM annotation, the highly conserved _453_PALPxK motif in Rap guanine nucleotide exchange factor 1 binds to the SH domain of Crk (53), the tyrosine of the _1068_PxPYAT motif conserved in roundabout homologue 1 from human to worm is phosphorylated by ABL kinase, suggesting a switchable binding site (54), and a PxWVxR motif in Mint1 mediates binding to calcium/calmodulin-dependent serine protein kinase (55).

It is worth noting that there are clear differences in the ability of the method to rediscover motifs within different classes of ELM. Modification (42.8%) and cleavage (19.4%) classes are returned at much lower efficiency than ligand (63.3%) and targeting (59.7%) classes. This is expected, as these sites, modification sites in particular, are well known to be evolutionarily plastic (56), and their differential conservation has been seen previously (9).

### SLiMPrints SLiM discovery on the human proteome

Of 20 253 human alignments, 18 212 had sufficient orthologues to run the SLiMPrints method. Approximately 368 million overlapping motifs were considered in a search space of 3 065 433 unmasked residues. To investigate the success of this SLiM discovery effort, the 172 motifs (in 168 different proteins) returned at *Sig*_motif_ ≤1^e^^−^^5^ were examined (Supplementary Table S3). On manual curation, 50 of these putative sites seemed to be the result of forced alignment of non-homologous positions in the alignment between highly divergent species. Twenty-three had experimental evidence for functionality, although only five are annotated in the ELM database (Table 1). The remaining 98 are of unknown function and have strong conservation compared with their surrounding residues (Supplementary Table S3). Greater than half (54) of the 98 motifs discovered in humans are identical in fly, 19 in worm and 5 in yeast. Approximately two-thirds (66) of these 98 motifs are also predicted as putative motifs by the ANCHOR motif discovery tool (mean ANCHOR probability: >0.5), which uses a complementary pairwise energy estimation approach (28). A full interactive list of the annotated results is available at http://bioware.ucd.ie/∼slimdb/SLiMPrints/.

**Table 1.** Highly significant hits (*Sig*_motif_ scores <1^e^*^−^*^5^) in the human proteome analysis with experimental evidence^a^

Protein name	Motif	Context	Function	Start	Reference
Stromal membrane-associated protein 2	DLLG	tapvm**DLLG**ldapv	Clathrin binding	185	(57)
AP-1 complex subunit γ-1	LLDL.G	sqand**LLDL**l**G**gndit	Clathrin binding	627	(58)
Dedicator of cytokinesis protein 4	PP.PP	gklis**PP**v**PP**rptqt	SH3 domain binding	1787	(59)
Tyrosine-protein kinase ABL1	AP.PP.R	kkkkt**AP**t**PP**k**R**sssfr	SH3 domain binding	610	(60)
Son of sevenless homologue 1	PPP.PP^b^	devpv**PPP**v**PP**rrrpe	SH3 domain binding	1149	(61)
Arrestin domain-containing protein 3	P.Y	rflpp**P**l**Y**seidp	WW domain binding	391	(62)
Enhancer of filamentation 1	D.YD.PR	vgsqn**D**a**YD**v**PR**gvqfl	SH2 domain binding	314	(63)
RAF proto-oncogene serine/threonine-protein kinase	RS.S.PN	lsqrq**RS**t**S**t**PN**vhmvs	14-3-3 binding	255	(64)
AP-3 complex subunit β-1	LLD.DD^b^	tkdvs**LLD**l**DD**fnpvs	Clathrin binding	817	(65)
Sorting nexin-33	W.DWDD	ddddd**W**d**DWDD**gctvv	Aldolase binding	116	(66)
Tyrosine-protein phosphatase non-receptor type 3	RS.S^b^	npamr**RS**l**S**vehle	14-3-3 binding	355	(67)
Rap guanine nucleotide exchange factor 1	PALP.K	qtdtp**PALP**e**K**krrsa	SH3 binding	453	(53)
Fez family zinc finger protein 2	FSI.IM	sktla**FSI**er**IM**aktse	Engrailed homology 1	29	(68)
Protein AF-9	KKR.K	eelsa**KKR**k**K**sssea	Nuclear localization signal	295	(69)
Serine/threonine-protein kinase LATS2	PPPPY	pdrrc**PPPPY**pkhll	WW domain binding	513	(70)
AP-1 complex subunit γ-like 2	LLDLL	sqlld**LLDLL**dgasg	Clathrin binding	625	(58)
Arrestin domain-containing protein 2	P.P.PP.Y	rlgal**P**er**P**ea**PP**e**Y**sevva	WW domain binding	332	(62)
Arrestin domain-containing protein 2	P.P	efryr**P**p**P**lysee	WW domain binding	383	(62)
Protein sprouty homologue 4	N.Y.D.P	tshve**N**d**Y**i**D**n**P**slalt	TKB domain binding	49	(71)
Cyclic adenosine monophosphate-dependent transcription factor ATF-6 alpha	R.LL	panqr**R**h**LL**gfsak	Cleavage site	415	(72)
Procollagen galactosyltransferase 2	EL	vpsrd**EL**	KDEL motif	624	(73)
Ubiquitin carboxyl-terminal hydrolase 8	P.DR.KK	siknv**P**qi**DR**t**KK**pavkl	SH3 binding motif	404	(74)
CCAAT/enhancer-binding protein beta	K.EP.E^b^	ppael**K**a**EP**gf**E**padck	Sumo site	173	(75)

^a^Context contains the motif and flanking regions of five residues, defined residues are bold and underlined. Start is the position of the motif in the protein.

^b^Denotes motifs annotated in ELM.

### SCF complex subunit F-box only protein 9

**Figure 6.**
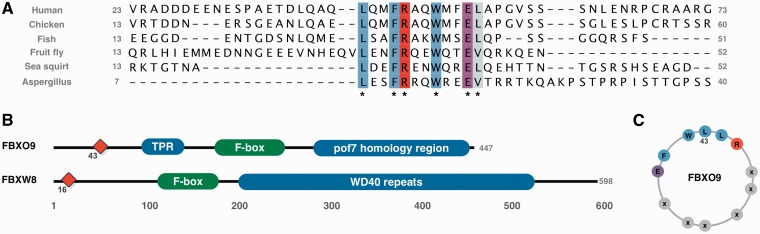
(**A**) Alignment of the 50 residues flanking the LxxFRxxWxxEL motif in FBXO9 orthologues showing the conservation across many diverse species, conserved residues are coloured by ClustalX colouring scheme. (**B**) Domain architecture of FBXO9 and FBXW8. Red diamond denotes position of LxxFRxxWxxEL motif. (**C**) Helical wheel representation of the LxxFRxxWxxEL motif.

A striking motif in the human proteome analysis was an _43_LxxFRxxWxxEL motif in FBXO9 (F-box only protein 9). The motif is conserved without degeneracy in any position from human to fungi (Figure 6A), although not present in plants. Motifs are often lost over large evolutionary distances, e.g. only 7.7% of human proteins are conserved in *Caenorhabditis elegans* (9); thus, for a motif of this length to be conserved without substitution over such a large range of species is unusual. The spacing of the conserved positions suggests a strong hydrophobic helical moment with a hydrophobic face (Leu^43^, Phe^46^, Trp^50^, Leu^54^), flanked by oppositely charged residues (Arg^48^, Glu^53^) (Figure 6C). ANCHOR (28), a method that quantifies the potential of a region to adopt secondary structure on binding, a strong indicator of a putative motif, also predicts the region to be functional (data not shown). A SLiMSearch analysis (23) of the human proteome returned only one other protein [F-box/WD repeat-containing protein 8 (FBXW8)] containing the motif (Figure 6B). Interestingly, FBXW8 and FBXO9 are not close homologues, they have different domain architectures, and several F-box containing proteins that are more closely related to FBXO9 lack the motif, even allowing for strong degeneracy conserving only physicochemical similarity ([Φxx[HYFW][KR]xx[FHYW]xx[DE]Φ). The function of the motif may relate to the protein’s role as the substrate-recognition component of the SCF (SKP1-CUL1-F-box protein)-type E3 ubiquitin ligase complex.

### Eukaryotic initiation factor eIF2A

**Figure 7.**
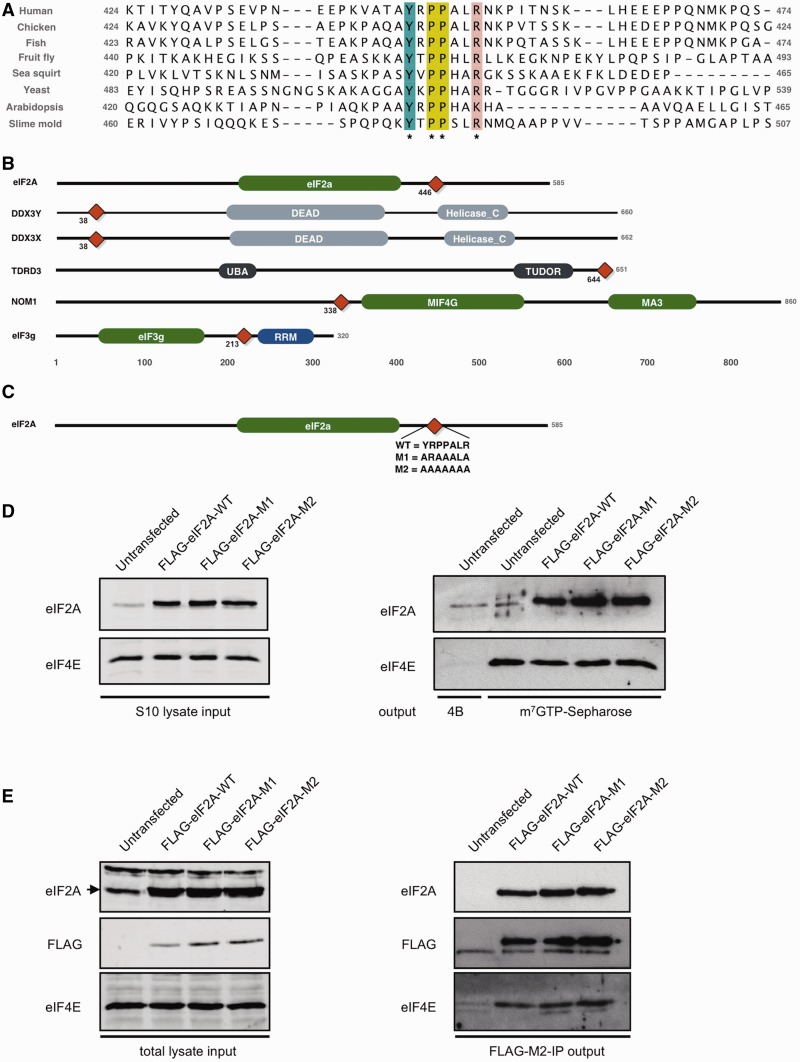
(**A**) Alignment of the 50 residues flanking the YxPPxLR motif in eIF2A orthologues showing the conservation across many diverse species, conserved residues are coloured by ClustalX colouring scheme. (**B**) Light grey boxes are domains involved in RNA metabolism, green domains are domains involved in translational regulation and grey domains have no obvious link to RNA processing. Red diamond denotes position of YxPPxLR motif. (**C**) Schematic of constructs used in assays with sequence variants shown. (**D**) Equal amounts of protein from S10 HeLa cell extracts were obtained after transfection with either no plasmid or wild-type or mutant forms of FLAG-tagged eIF2A and were subjected to SDS–PAGE and immunoblotting with the antibodies indicated (left hand panels). The extracts were then subjected to m^7^GTP Sepharose chromatography (right hand panel, lanes 2–5) to recover proteins associated with eIF4E. Untransfected cell extract was also incubated with control 4B Sepharose resin (lane 1). (**E**) Extracts from HeLa cells transfected as described in panel D were subjected to co-immunoprecipitation as described in ‘Materials and Methods’ section with AminoLink agarose resin coupled to FLAG-M2 antibody. Immunoblotting of proteins from the total cell extract (left hand panels) or eluted proteins (right hand panels) was carried out using the antibodies indicated.

Another notable result was the _446_AYxPPxxR motif discovered in human translation initiation factor eIF2A (Q9BY44; *Sig*_motif_ <1^e^^−^^5^), a transfer RNA (tRNA)-binding protein thought to function in a translation initiation pathway independent of the ternary complex which contains eIF2, GTP and the initiating Met-tRNA_i_ (76). This bears a striking similarity to the YxPPxΦR motif that mediates binding of the adenosine triphosphate (ATP)-dependent RNA helicase DDX3X to a key initiation factor, eIF4E (77) (ELM LIG_eIF4E_2). It should be noted that these motifs were only added to the ELM database (22) since the ELM benchmarking exercise. The YxPPxx[KR] motif in human eIF2A is conserved in fungi, plants and amoeba (Figure 7A), indicating that it might have an evolutionarily ancient functional relevance, as befits a protein involved in a core process like protein synthesis. Using data from the eIF2A alignment (Figure 7A) and LIG_eIF4E_1, we performed a SLiMSearch (23) analysis of YxPPx[ILMVA][KR] instances in the human proteome: all highly significant hits (*Sig*_motif_ <0.001) occur in messenger RNA (mRNA) translation-related proteins (Table 2), with the exception of carnitine deficiency-associated gene expressed in ventricle 3 (CDV3), which has no known function. Specifically, returned proteins are known components of eIF complexes (eIF3g, eIF2A) or known to be direct binders of eIF-related proteins [ATP-dependent RNA helicase DDX3X, ATP-dependent RNA helicase DDX3y, nucleolar MIF4G domain-containing protein 1, (NOM1), Tudor domain-containing protein 3 (TDRD3)]. With the exception of the divergent instances in DDX3X and DDX3Y, all instances seem to have evolved convergently having different domain architecture and no obvious homology outside the motif (Figure 7B).

**Table 2.** Significant SLiMsearch hits (*Sig*_motif_ <0.001) for the YxPPx[ILMVA][KR] regular expression^a^

Gene	Protein	Context	Position
eIF2A	Eukaryotic translation initiation factor 2A	kvata**Y**r**PP**a**LR**nkpit	457
DDX3X	ATP-dependent RNA helicase DDX3X	askgr**Y**i**PP**h**LR**nreat	49
DDX3Y	ATP-dependent RNA helicase DDX3Y	askgr**Y**i**PP**h**LR**nreas	49
TDRD3	Tudor domain-containing protein 3	ptqqf**Y**q**PP**r**AR**n	650
NOM1	Nucleolar MIF4G domain-containing protein 1	gsgek**Y**i**PP**h**VR**qaeet	349
CDV3	Protein CDV3 homologue	mtsgv**Y**r**PP**g**AR**ltttr	180
ZC2HC1A	Protein FAM164A	srtqv**Y**k**PP**a**LK**ksnsp	180
eIF3g	Eukaryotic translation initiation factor 3 subunit G	nktgk**Y**v**PP**s**LR**dgasr	224

^a^Context contains the motif and flanking regions of five residues, defined residues are bold and underlined. Start is the position of the motif in the protein.

During translation in eukaryotes, a number of subcomplexes of eukaryotic initiation factors (eIFs) assemble into a preinitiation complex, bringing together the mRNA with a small (40S) ribosomal subunit [reviewed in (78)]. Key to this process is the m^7^GTP cap structure present at the 5′ end of all mRNAs, which is bound by eIF4E during the process of cap-dependent translation. This protein resembles a cupped hand, with the internal surface used for interactions with the cap structure (79). The dorsal surface of the protein acts as a site of interaction with a number of other proteins, which compete with each other to form complexes that are either competent for translation (e.g. when the scaffold proteins eIF4GI or eIF4GII bind, forming the eIF4F complex, and recruit the 40S subunit) (80) or inhibitory (e.g. when 4E-BPs 1–3 bind, preventing the recruitment of other proteins) (81). The LIG_eIF4E_1 motif, YxxxxLΦ, is responsible for this competitive complex formation in the eIF4E-interacting proteins eIF4GI, eIF4GII and the 4E-BPs (82), and the YxPPxΦR motif of DDX3X is thought to competitively bind at the same site (77). These results identify potential novel eIF4E-binding partners, which could play important roles in initiation regulation, although we cannot rule out the possibility that there is a second initiation-related protein that recognizes the same motif.

To explore this possibility further, we investigated whether the eIF2A and eIF4E proteins interact. Before this work, no protein interaction was known, although a genetic interaction has been reported in yeast, with an eIF2A/eIF4E knockout strain arresting at the G_2_/M border (83). We investigated the potential interaction of eIF4E and eIF2A in HeLa cells. Our initial work to co-immunoprecipitate the endogenous eIF2A protein from HeLa cells showed that eIF2A and eIF4E could be detected after elution from resin cross-linked to an eIF2A antibody, and neither protein bound to an agarose resin control (data not shown). These results indicate that eIF4E does indeed interact with eIF2A, either directly or as part of the same complex, as supported by m^7^GTP-Sepharose chromatography and co-immunoprecipitation using an anti-FLAG antibody (Figure 7). It should be noted that a small amount of eIF2A is detected in the elution from the 4B control Sepharose resin (Figure 7D), suggesting there is some intrinsic ability of this protein to bind non-specifically to the resin. However, in the reciprocal immunoprecipitation experiment, eIF4E only elutes with FLAG-tagged eIF2A proteins (Figure 7E), confirming that the interaction between the two proteins is genuine. Although functionally important, the eIF4E ligand may not be necessary for binding: surface plasmon resonance has identified additional sites essential for the eIF4E:-eIF4G interaction (SDVVL) (84) and the eIF4E:-4E-BP interactions (PGVT[ST]) (85), whereas the C-terminal domain of DDX3X (DDX3^3536–3661^) was shown to weakly interact with eIF4E despite the YxPPxΦ[KR] motif being N-terminal (DDX3^38–44^) (77). Therefore, we investigated whether the motif was sufficient for eIF4E binding in eIF2A using mutations at the potential eIF4E binding site, from YRPPALR to either ARAAALA (M1) or AAAAAAA (M2), transfected into HeLa cells (Figure 7C). The binding is not abrogated when the proposed site of interaction is mutated. Thus, as with other 4E-binding proteins, interaction of the two proteins is not dependent on the motif in eIF2A, and we speculate that eIF2A has a second site of interaction. Although not necessary for binding, point mutations in the DDX3X YxPPxΦ[KR] motif were shown to impair its regulatory activity (77). Given this, we propose that the observed eIF2A-eIF4E interaction and the strong evolutionary conservation of the YxPPxx[KR] motif in eIF2A is highly suggestive that the motif is involved in regulation of eIF4E activity, even if it may not be necessary and/or sufficient for binding to eIF4E, and it should be the focus of further study. As eIF2A is able to supply Met-tRNAi to the 40S ribosome in a GTP-independent manner (86), how it may function in concert with the wider initiation factor machinery is of particular interest. For instance, this new interaction may allow the formation of a cap-dependent translation initiation complex that does not require the usual eIF4G scaffold proteins or the eIF2-containing ternary complex.

### Challenges for using conservation in SLiM discovery

Intrinsically disordered regions of proteins have much greater evolutionary fluidity than globular regions (48), as the same property of disorder may be maintained by sequences undergoing regular mutation, insertions and deletions. Yet, it is clear that functional motifs contained within these regions are, in general, more evolutionarily conserved than surrounding residues (30), a fact that has already been exploited in the identification of novel instances of previously known SLiMs (23,34,87) and in the discovery of novel motif classes (35). This rationale is further supported by recent studies in mononegavirales, which used profile–profile comparison (39), and in yeast, which used a phylogenetic hidden Markov model (40), to identify conserved residues versus the background conservation of disordered protein regions in an analogous approach.

Many issues confound the ability of the SLiMPrints method to return functional motifs. Methodologically, multiple alignment tools are not designed to align disordered regions (49), often misaligning short conserved regions and forcing the alignment of regions that lack common evolutionary descent. Alignment might be further impaired by potential issues arising from splice variation and incomplete sequencing/annotation of some species. Large gaps in species coverage compound this issue further, especially (in the case of the human analysis) at the vertebrate/invertebrate boundary. Ultimately, this introduces noise and loss of signal, and subsequently negatively affects the quality of the conservation metric. Indeed, in this study, we found 50 of the 172 highest ranking motifs to be affected by alignment error. Improvements to species coverage, alignment tools and conservation metrics, in conjunction with the development of accurate methods to successfully recognize poorly aligned residues in disordered regions, will drastically improve the ability of the next generation of conservation-based motif discovery tools. Users of SLiMPrints, or of any conservation approach to identify motifs, need to pay careful attention to the quality of the sequence annotation/protein prediction, alternative splicing and other factors that can influence interpretation considerably.

The SLiMPrints method also has a clear hypothesis, searching for strong islands of conservation in a disordered sea of evolutionary drift. A large proportion of motifs simply do not exhibit this characteristic. Numerous motifs occur in regions containing multiple overlapping motifs, regulatory modification sites and disordered domains that create an extended region of many conserved residues (9,10). Motifs are also gained and lost relatively quickly (on an evolutionary timescale), and, as a result, motifs often do not have a level of conservation that is indicative of a strong functional constraint. However, despite these issues, there is clearly a strong signal in the evolutionary constraint of many functional motifs.

Because of the issues highlighted previously, the SLiMPrints method is not capable of discovering all functional motifs in a proteome, but rather to highlight interesting putative motifs, identifiable by their conservation pattern. The method should excel in the identification of the ‘low-hanging fruit’ of easily identified motifs. As such, it will prove valuable for the identification of protein motifs of likely functional importance in lesser-studied proteins, giving experimentalists a starting point to investigate the functionality of these proteins. Other methods designed for *de novo* identification of motifs from primary sequence, such as α-MoRF (27), ANCHOR (28) and SLiMPred (29), are complementary to the SLiMPrints approach. As each of these approaches recognize putative functionality based on different hypotheses (supported by observed attributes of functional motifs), future methods that incorporate the best features of each method should show stronger overall performance.

## CONCLUSIONS

We have shown that novel motif classes can be discovered using a conservation-based metric and anticipate that application of the statistical framework described in this article will aid in the systematic identification of functional modules in disordered regions, particularly for poorly annotated proteomes. SLiMPrints represents a useful addition to the growing toolkit of bioinformatics methods for motif discovery that simplify and accelerate the process of documenting regions of potential interest, guide experimental discovery of novel SLiMs and enrich our current knowledge of protein interaction interfaces within intrinsically disordered regions. Furthermore, the whole proteome search for putative functional human SLiMs has provided a valuable resource for further experimental validation.

## SUPPLEMENTARY DATA

Supplementary Data are available at NAR Online: Supplementary Tables 1–3.

## FUNDING

EMBL Interdisciplinary Postdoctoral fellowship (to N.E.D.); Science Foundation Ireland grant [08/IN.1/B1864 to D.C.S.]; Biotechnology and Biological Sciences Research Council (BBSRC) New Investigator Award [BB/H006834/1 to M.J.C., J.L.C. and BB/I006230/1 to R.J.E.]. Funding for open access charge: EMBL.

*Conflict of interest statement*. None declared.

## Supplementary Material

Supplementary Data
