# Supplementary material for: SLiMPrints: conservation-based discovery of functional motif fingerprints in intrinsically disordered protein regions
Source: Nucleic Acids Res. 2012 Sep 12;40(21):10628–41. doi: 10.1093/nar/gks854 (PMC3510515; doi:10.1093/nar/gks854)
Supplement: Supplementary Data [file supp_40_21_10628__index.html]

SLiMPrints: conservation-based discovery of functional motif fingerprints in intrinsically disordered protein regions — SLiMPrints: conservation-based discovery of functional motif fingerprints in intrinsically disordered protein regions — Supplementary Data 

# SLiMPrints: conservation-based discovery of functional motif fingerprints in intrinsically disordered protein regions

## Supplementary Data

files

**Files in this Data Supplement:**

- Supplementary Data - csv file
- Supplementary Data - csv file
- Supplementary Data - csv file
